# Proteinuria, Hypoalbuminemia, and Chronic Lymphocytic Leukemia: An Unusual Trio

**DOI:** 10.1177/2324709618764207

**Published:** 2018-03-14

**Authors:** William Wung, Shubha Ananthakrishnan, Brian A. Jonas

**Affiliations:** 1 University of California, Davis, Sacramento, CA, USA

**Keywords:** chronic lymphocytic leukemia, nephrotic syndrome, venous thromboemboli

## Abstract

Chronic lymphocytic leukemia (CLL) is a chronic, progressive lymphoproliferative disorder characterized by a monoclonal population of functionally incompetent lymphocytes. Renal involvement is rare and poorly described. A 57-year-old male with no prior medical history was diagnosed with CLL and followed with a watch and wait approach. He was referred to our institution several months later due to concern for Richter’s transformation to diffuse large B-cell lymphoma. A positron emission tomography/computed tomography scan showed no evidence of diffuse large B-cell lymphoma; however, the patient was noted to have hypoalbuminemia, nephrotic range proteinuria, an acute left renal vein thrombus, and a right pulmonary embolus. A nephrotic syndrome workup including autoimmunity and infection was unremarkable, and a kidney biopsy was deferred due to concern for renal compromise in the setting of a renal vein thrombus. The patient was treated with 6 cycles of reduced-dose fludarabine, cyclophosphamide, and rituximab for a presumed CLL-associated nephrotic syndrome and anticoagulation for his venous thromboemboli. At 6-month follow-up, the patient achieved complete remission of his CLL with normalization of all cell lines and resolution of his nephrotic range proteinuria. Repeat computed tomography scans showed no evidence of recurrent venous thromboemboli. This case demonstrates a potential role of empiric chemotherapy in cases of CLL-associated nephrotic syndrome given its potentially life-threatening sequelae and response to treatment.

## Introduction

Chronic lymphocytic leukemia (CLL) is a progressive, lymphocytic disorder characterized by accumulation of immature, functionally incompetent lymphocytes. Staging is typically done via the Rai and Binet systems, both of which are based on the presence and degree of disease-related anemia, thrombocytopenia, and organomegaly (Tables 1 and 2). Treatment with chemoimmunotherapy or small molecular inhibitors is typically indicated for high-risk disease (eg, Binet stage C or Rai stage III/IV) and select cases of intermediate disease (Binet stage B or Rai stage I/II).^1^ Renal involvement such as nephrotic syndrome is rare and poorly described.^2^ We present a case of a 56-year-old male with CLL who presented with nephrotic range proteinuria and venous thromboemboli, both of which subsequently resolved with treatment of his CLL.

**Table 1.** Binet Clinical Staging for Chronic Lymphocytic Leukemia^1^.

Stage	Description
A	One to 2 enlarged lymphoid areas (eg, cervical, axillary, inguinofemoral, spleen, liver)
B	Thee or more enlarged lymphoid areas
C	Presence of anemia (hemoglobin <10.0 g/dL) or thrombocytopenia (platelets <100 000/µL)

**Table 2.** Modified Rai Clinical Staging for Chronic Lymphocytic Leukemia^1^.

Risk	Stage	Description
Low	0	Lymphocytosis (eg, lymphoid cells >30% cells in blood or marrow)
Intermediate	I	Lymphocytosis and lymphadenopathy
	II	Lymphocytosis with enlarged spleen or liver, with or without lymphadenopathy
High	III	Lymphocytosis with anemia (hemoglobin <11.0 g/dL), with or without enlarged spleen, liver, or lymph nodes
	IV	Lymphocytosis with thrombocytopenia (platelets <100 000/µL)

## Case Report

A 57-year-old male with no prior medical history presented to an outside hospital with progressive fatigue, decreased exercise tolerance, and lower extremity edema of 3 months duration. No lymphadenopathy or hepatosplenomegaly was noted on exam. Laboratory tests were significant for a leukocytosis of 95 200/µL with 84% lymphocytes, smudge cells on peripheral smear, hemoglobin 13.2 g/dL, platelet count 177 000/µL, creatinine 1.2 mg/dL, serum albumin 2.1 g/dL, and hemoglobin A1c of 5.7% (Table 3). No prior laboratory tests were available for comparison, as the patient had not seen a physician in several years. Peripheral blood immunophenotyping showed a monoclonal population of cells positive for CD5, CD19, CD20, CD22, and CD23 and negative for CD38, an immunophenotype that is consistent with CLL. Peripheral blood fluorescent in situ hybridization was positive for trisomy 12 and negative for del(11p), del(13q), and del(17q). ZAP70 and immunoglobulin heavy-chain variable-region mutations were also negative. Serum β-2 microglobulin was elevated at 3.7 µg/mL (normal 0-3 µg/mL). The patient was diagnosed with Rai stage 0 CLL and was followed with a watch and wait approach until approximately 6 months afterward when a computed tomography (CT) scan reportedly showed a left lower quadrant mass concerning for possible Richter’s transformation to diffuse large B-cell lymphoma. He was subsequently referred to our institution for further evaluation.

**Table 3.** Summary of Laboratory Tests.

Parameter	Six Months Prior to Admission	On Admission	Prior to Chemotherapy	Six Months After Chemotherapy
White blood cell count (normal 4500-11 000/mm^3^)	95.2	77.0	72.0	4.3
Hemoglobin (normal 13.5-17.5 g/dL)	13.2	11.5	10.7	13.9
Platelet count (normal 13 000-400 000/mm^3^)	177	174	178	174
Creatinine (normal 0.44-1.27 mg/dL)	1.2	1.3	1.24	0.77
Albumin (normal 3.5-5.5 g/dL)	2.1	1.4	1.4	3.8
Lactate dehydrogenase (normal 90-200 U/L)	322	—	242	—

**Figure 1.**
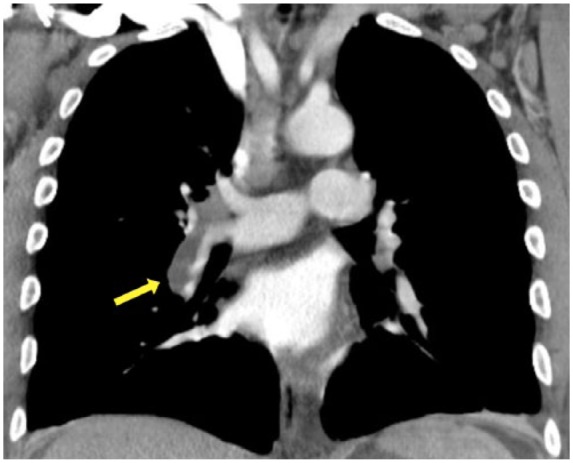
Computed tomography scan of chest with contrast. Large acute pulmonary embolus involving the right pulmonary artery extending into the interlobar artery and its branches (yellow arrow).

**Figure 2.**
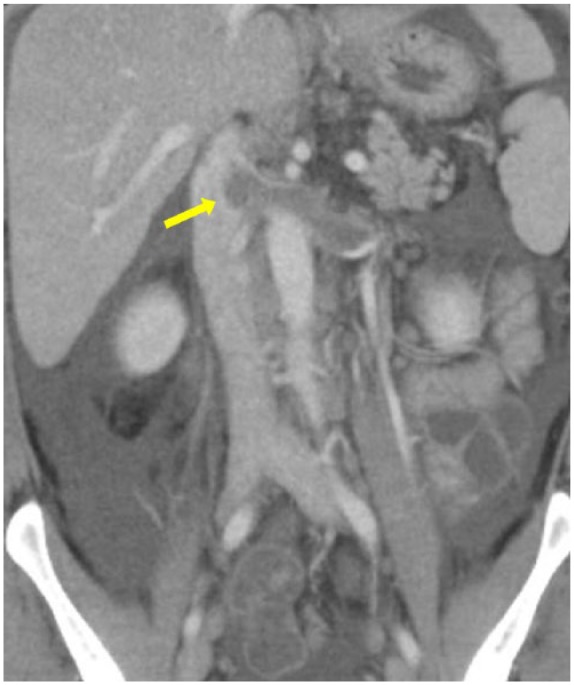
Computed tomography scan of abdomen/pelvis with contrast. Left renal vein thrombus with total luminal occlusion and extension into the inferior vena cava (yellow arrow), along with diffuse lymphadenopathy (periportal, mesenteric, retroperitoneal), hepatomegaly, and splenomegaly.

On admission, physical exam was significant for bilateral axillary and inguinal lymphadenopathy, hepatomegaly, splenomegaly, and diffuse anasarca with pronounced scrotal edema. Laboratory tests were notable for a leukocytosis 77 000/µL with 93% lymphocytes, hemoglobin 11.5 g/dL, platelet count 174 000/µL, creatinine 1.3 mg/dL, estimated creatinine clearance (CrCl) of 95 mL/min, and serum albumin 1.4 g/dL (Table 3). Platelets, serum electrolytes, transaminases, alkaline phosphatase, total bilirubin, and international normalized ratio were within normal limits. Repeat peripheral blood flow cytometry confirmed CLL with a monoclonal population of cells with the immunophenotype previously noted along with negative expression of CD34, CD117, and FMC7. Urinalysis showed 3+ protein, and a 24-hour urine protein collection showed 11 g/24 h (normal <150 mg/24 h). The high-grade proteinuria, hypoalbuminemia, and anasarca confirmed the diagnosis of nephrotic syndrome. The patient also underwent a whole-body positron emission tomography/CT scan to evaluate for possible Richter’s transformation. Diffuse mesenteric, inguinal, and retroperitoneal lymphadenopathy was noted consistent with Rai Stage II, but there was no evidence of hypermetabolic masses concerning for diffuse large B-cell lymphoma. However, an acute right pulmonary embolus (Figure 1) and a left renal vein thrombus (Figure 2) were noted on CT. The patient was started on low-molecular-weight heparin while undergoing further evaluation for his nephrotic-range proteinuria.

Antinuclear antibody, HIV, hepatitis B/C, and phospholipase-A2 receptor antibody titers were negative. Serum and urine protein electrophoresis showed decreased and increased total protein, respectively, but were otherwise unremarkable. Serum κ and λ light chains were within normal limits, and no urine Bence-Jones proteins, dysmorphic cells, or casts were detected. A kidney biopsy was deferred due to concern for risk of bleeding in the setting of renal vein thrombosis and anticoagulation. Therefore, it was concluded that the patient’s nephrotic syndrome and venous thromboemboli, a known complication of nephrotic syndrome, were due to CLL given his otherwise unremarkable nephrotic syndrome workup. He was started on 6 cycles of reduced-dose fludarabine, cyclophosphamide, and rituximab (FCR-Lite) chemotherapy and continued on low-molecular-weight heparin.^3^ At 6-month follow-up, the patient achieved complete remission of his CLL with normalization of all cell lines, serum creatinine, hypoalbuminemia, and nephrotic range proteinuria (Table 3). No lymphadenopathy was noted, and repeat CT scans showed no evidence of recurrent venous thromboemboli.

## Discussion

Kidney involvement is an uncommon but potentially serious sequela of CLL, can occur at any stage of CLL, and should be considered as a leading diagnosis once other causes of renal impairment have been considered. Membrano-proliferative glomerulonephritis (MPGN) is the most common cause of CLL-associated renal involvement, with CLL-cell infiltration, thrombotic microangiopathy (TMA), and minimal change disease as other notable etiologies.^4^ Cases of CLL-associated focal segmental glomerulosclerosis have been reported as well.^5^

Evaluation involves a thorough history and physical exam, urinalyses, and serologies to evaluate for other causes of nephrotic and/or nephritic syndromes, for example, autoimmunity, infection, and so on. Anti-phospholipase A2 receptor antibodies are often used in evaluation of idiopathic membranous nephropathy; however, their role in CLL-associated renal impairment is less well established.^6,7^ Diagnosis often ultimately entails a renal biopsy in order to differentiate among the different etiologies of kidney damage.^1,5^ This is especially important given the differences in management and prognosis. For instance, several cases of renal TMA diagnosed on biopsy resolved after discontinuation of chemotherapy, in particular pentostatin, that itself has been associated with TMA.^4^ Conversely, CLL-associated MPGN appears to respond well to alkylating agents, in particular cyclophosphamide, especially given their existing use in treating idiopathic MPGN.^4^

It is unclear if any specific CLL stage, mutation, or serologic marker predisposes to a higher or lower risk of CLL-associated kidney injury. The Rai and Binet staging systems are typically used to determine prognosis and/or decision to initiate treatment. However, these are based on physical exam and complete blood count, and do not include existing or future risk of kidney function/impairment. Trisomy 12 and del(13q) have been associated with favorable prognoses (eg, progression-free survival, response to treatment, etc), while del(11q), del(17q), CD38, immunoglobulin heavy chain variable region gene mutations, and elevated β-2 microglobulin levels have been associated with poor prognoses.^5^ However, a single-center case series of 49 patients with biopsy-proven CLL-associated renal injury including nephrotic syndrome showed no predominant mutations, immunophenotypes, or serologic markers.^4^ Another multicenter case series of 15 similar patients was also inconclusive.^8^ Further work such as a systematic review with a larger sample size may elucidate further insight into potential genetic and/or serologic risk factors of CLL-associated renal injury.

Kidney involvement also has potential implications toward CLL treatment. Currently, there is no standard regimen and/or agent(s) for treatment of symptomatic CLL. Specific agents include purine analogs (eg, fludarabine, pentostatin), alkylating agents (eg, chlorambucil, cyclophosphamide, bendamustine), monoclonal antibodies (eg, rituximab), and Bruton’s tyrosine kinase inhibitors (eg, ibrutinib). Most young (<70 years), previously untreated patients with intact renal function are treated with fludarabine-based therapy including cyclophosphamide and/or and rituximab (FCR and FR, respectively), as these regimens have demonstrated superior response rates and progress-free survival in this patient population when compared with other regimens (eg, chlorambucil-based).^3^ However, CrCl is one of the strongest predictors of fludarabine toxicity, as such non-fludarabine regimens are often chosen in patients with an estimated CrCl <80 mL/min.^9^ Bendamustine plus rituximab appears to be slightly less effective but with similar overall survival rates and less toxic effects when compared with FCR.^10^ Chlorambucil is also sometimes used for cases of CLL with renal impairment, but this has shown to be inferior to fludarabine- and bendamustine-based reigmens.^3,11^

In the case of our patient, his profound anasarca, hypoalbuminemia, proteinuria, venous thromboemboli, normal transaminases, and international normalized ratio were suggestive of an underlying nephrotic syndrome. However, his workup for autoimmunity, infection, and other common causes of nephrotic range proteinuria was unrevealing. Therefore, we attributed his nephrotic syndrome to his known CLL as a diagnosis of exclusion. We discussed a potential kidney biopsy with multiple consulting teams to further elucidate the mechanism of kidney injury. However, this was deferred given that (1) his left kidney was already compromised by his left renal vein thrombus and (2) risk of acute renal failure if his right-kidney were to be injured during said biopsy. Therefore, we decided to initiate chemoimmunotherapy despite our patient having Rai stage II CLL given that there were no other explainable causes of nephrotic syndrome. We chose FCR because of its superiority over non-fludarabine regimens in young, previously untreated patients such as ours, and because his CrCl was well over 80 mL/min at time of chemotherapy initiation. Although current guidelines do not include CLL-associated nephrotic syndrome as an indication for treatment, we felt the benefits outweighed the risk in this situation especially given his multiple complications.

Our case shows that CLL can cause nephrotic syndrome in the absence of other etiologies. We also demonstrate that a renal biopsy, although helpful, may not always be needed in such cases. Our case also shows that CLL-associated nephrotic syndrome may be an indication for CLL treatment when no other indications exist, in particular with FCR chemoimmunotherapy in cases without severe renal impairment. To our knowledge, this is the only reported case of CLL-associated nephrotic syndrome that resolved with FCR. It is unknown if newer agents (eg, ibrutinib, idelaisib, ventoclax) or monoclonal antibodies would have the same effect.
